# Identification of a *Coxiella burnetii* outer membrane porin required for intracellular replication

**DOI:** 10.1128/iai.00448-24

**Published:** 2025-03-12

**Authors:** Zi Yang, Jeffrey K. Duncan-Lowey, Craig R. Roy

**Affiliations:** 1 Department of Microbial Pathogenesis, Yale University School of Medicine 198940 , New Haven, Connecticut, USA; University of Pennsylvania Perelman School of Medicine, Philadelphia, Pennsylvania, USA

**Keywords:** *Coxiella*, intracellular bacteria, porins, secretion systems

## Abstract

*Coxiella burnetii* is a gram-negative, obligate intracellular pathogen that causes human Q fever. Within host cells, *C. burnetii* proliferates in a spacious, acidic, lysosome-derived *Coxiella*-containing vacuole (CCV) by a process that requires the Dot/Icm type IVB secretion system to deliver effectors that manipulate host cell functions. A previous transposon mutagenesis screen identified the gene *cbu0937* as being important for intracellular replication of *C. burnetii*. Here, the function of Cbu0937 was investigated. The *cbu0937*::Tn mutant had no detectable defect replicating in the axenic acidified citrate cysteine medium 2. Additionally, intracellular replication of the *cbu0937*::Tn mutant was not restored by co-infection of host cells with an isogenic wild-type strain of *C. burnetii*. Thus, the *cbu0937*::Tn mutant has a cell-intrinsic intracellular replication defect. Intracellular replication of the *cbu0937*::Tn mutant was restored by complementing the gene *in trans* with a plasmid encoding either untagged or an epitope-tagged version of Cbu0937. Analysis of the predicted structure of the Cbu0937 protein using AlphaFold revealed high similarity between Cbu0937 and several bacterial porins. Fractionation studies and surface labeling of *C. burnetii* producing a functional epitope-tagged protein indicated the localization of Cbu0937 to the bacterial outer membrane. From these data, we conclude that *cbu0937* encodes a porin that plays an essential role in supporting *C. burnetii* intracellular replication, which likely involves the acquisition of an important metabolite in the CCV lumen.

## INTRODUCTION

*Coxiella burnetii* is the etiologic agent of Q fever (1, 2), a zoonotic disease that presents in both acute and chronic forms. Acute Q fever typically manifests as a flu-like illness (3). A small percentage of cases progresses to chronic Q fever, which can lead to severe illness such as valvular endocarditis, hepatitis, and other life-threatening syndromes (4). Q fever most often occurs in people who are in contact with farm animals (5). The most common route for transmission is the inhalation of aerosols containing *C. burnetii*, shed from birth products, urine, feces, and milk of infected animals (6).

Once inhaled, *C. burnetii* can infect various types of cells in the alveolar space, with alveolar macrophages serving as the main host cells for infection (7). As a gram-negative, obligate intracellular pathogen, *C. burnetii* employs a sophisticated mechanism to establish a niche within host cells. After being internalized, *C. burnetii* initially resides in the phagosome that fuses with endocytic organelles, mediating the delivery of bacteria to an acidified lysosome (8). The ability to establish and replicate within the unique lysosome-derived vacuole, known as the *Coxiella*-containing vacuole (CCV), is a distinctive feature of *C. burnetii* pathogenesis. The acidic environment is essential for activating *C. burnetii* metabolism and initiating the translocation of bacterial effector proteins by the Dot/Icm type IVB secretion system (9, 10). *C. burnetii* effectors are critical for subverting host cellular processes, including the regulation of vesicular trafficking (11, 12), influencing autophagy (13–16), modulation of host immune responses (17, 18), inhibition of cell death pathways (19–22), and manipulation of various other eukaryotic cell functions (23–25) to facilitate bacterial survival and replication.

A screen of a *C. burnetii* effector mutant sublibrary for deficiencies in CCV biogenesis and intracellular replication in HeLa cells revealed a severe intracellular replication defect of a *cbu0937* transposon insertion mutant (*cbu0937*::Tn) (26). A comparable intracellular replication defect was also reported in another study utilizing an independently constructed *cbu0937* mutant (27). The *cbu0937* gene encodes a hypothetical protein that had been shown to interact with DotF in bacterial two-hybrid assays and was proposed to function as a Dot/Icm effector based on studies examining translocation of the protein into host cells by *Legionella pneumophila* (28). Ectopic production of the Cbu0937 protein in eukaryotic host cells revealed localization of Cbu0937 to the mitochondrial network, and endogenously produced Cbu0937 was detected in a mitochondria fraction isolated from *C. burnetii*-infected cells (29). Thus, it was proposed that Cbu0937 functions as a mitochondria-localized effector. Here, we set out to investigate the function of Cbu0937 to better understand how this protein contributes to *C. burnetii* intracellular replication.

## RESULTS

### The gene *cbu0937* is important for intracellular replication of *C. burnetii*

**Fig 1:**
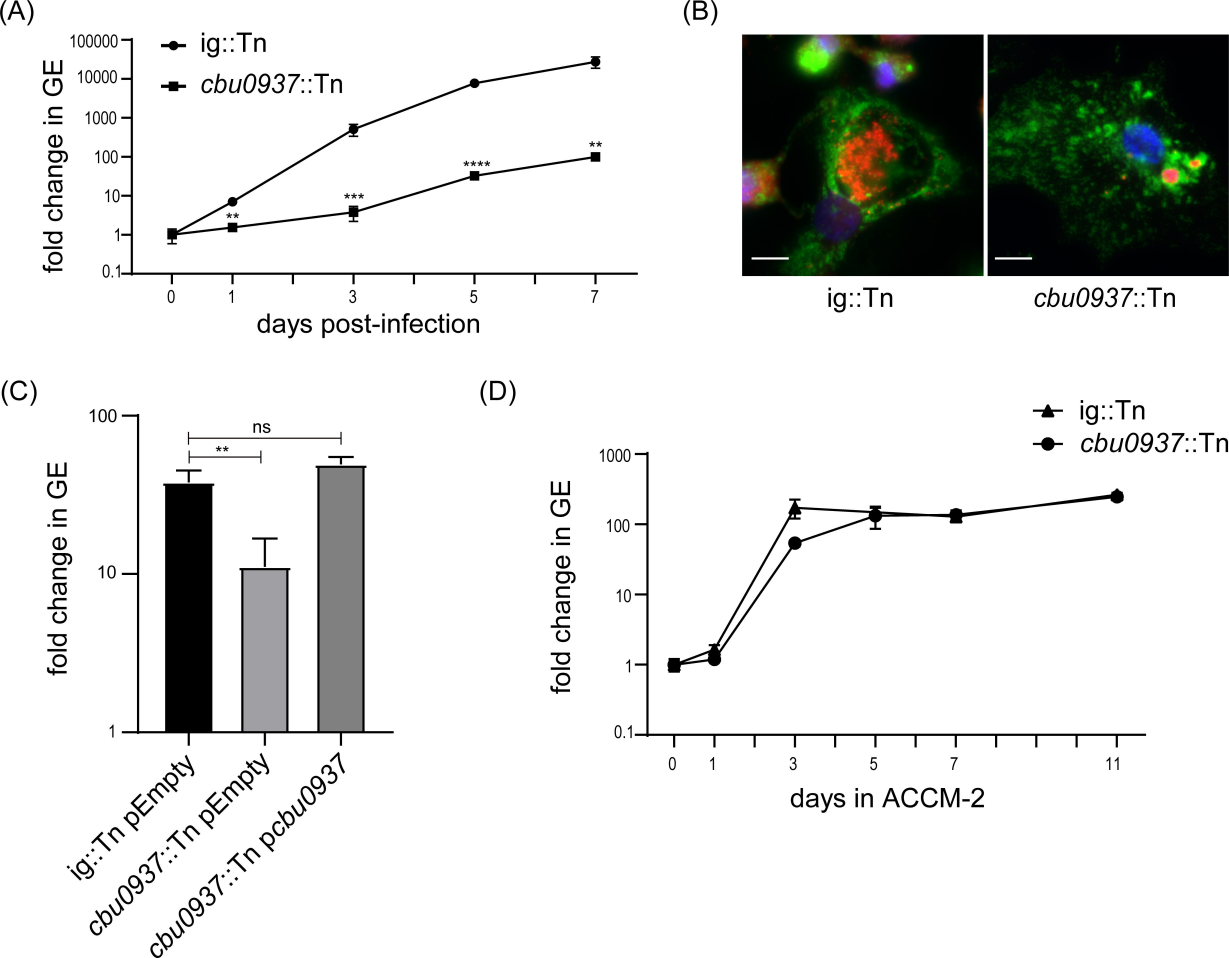
The gene *cbu0937* is important for intracellular replication of *C. burnetii*. (A) Intracellular replication over 7 days in THP-1 cells of the *cbu0937*::Tn mutant and the ig::Tn strain quantified by GE. The data shown represent one experiment from three independent experiments, each performed with three biological replicates. For each sample, quantitative PCR (qPCR) was performed in duplicate. Error bars are ± standard deviation. Statistical significance was determined using Student’s *t*-test. Represented *P*-values are relative to the strain ig::Tn. **, *P* < 0.01; ***, *P* < 0.001; ****, *P* < 0.0001. (B) Representative immunofluorescence micrographs of THP-1 cells infected with the *cbu0937*::Tn mutant or the ig::Tn strain for 5 days at a multiplicity of infection (MOI) of 2. Fixed cells were stained with anti-LAMP-1 antibody (green), anti-*C*. *burnetii* antibody (red), and 4′,6-diamidino-2-phenylindole (DAPI, blue). Scale bars = 10 µm. (C) Intracellular replication quantified by GE of the ig::Tn strain harboring an empty vector, the *cbu0937*::Tn mutant harboring an empty vector, and the *cbu0937*::Tn mutant harboring a *cbu0937* complementation plasmid after infection of THP-1 cells for 5 days at an MOI of 2. The data shown represent one experiment from three independent experiments, each performed with three biological replicates and two technical replicates. Error bars are ± standard deviation. Analysis of variance was used to compare samples. **, *P* < 0.01; ns, not significant. (D) Replication over 11 days in ACCM-2 of the *cbu0937*::Tn mutant and the ig::Tn strain. Replication was quantified by fold change in GE. The data shown represent one experiment from three independent experiments, each performed with three biological replicates and two technical replicates.

Previous studies reported that *cbu0937*::Tn mutants exhibited deficiencies in intracellular replication (26, 27). To verify these findings, we infected THP-1 macrophage-like cells with a *cbu0937*::Tn mutant and a *C. burnetii* Nine Mile phase II (NMII) control strain having a transposon insertion in a neutral intergenic region of the chromosome (ig::Tn). Genome equivalents (GEs) of the corresponding strains were quantified to assess intracellular replication, and immunofluorescence microscopy was used to determine CCV sizes. Compared to the control strain, the *cbu0937*::Tn mutant displayed significantly impaired replication in THP-1 cells over 7 days, as demonstrated by substantially reduced GEs (Fig. 1A). Consistent with the intracellular replication defect, the *cbu0937*::Tn mutant formed very small lysosomal-associated membrane protein 1 (LAMP-1)-positive CCVs at 5 days post-infection (Fig. 1B; Fig. S1). This intracellular replication defect of the *cbu0937*::Tn mutant was also observed in HeLa cells (Fig. S2). Expression of wild-type *cbu0937* from a plasmid complemented the intracellular replication defect in the *cbu0937*::Tn mutant (Fig. 1C), which demonstrates that the observed intracellular replication defect is due to the loss of *cbu0937* function. Importantly, both the *cbu0937*::Tn mutant and the isogenic ig::Tn strain replicated similarly over 11 days in axenic acidified citrate cysteine medium 2 (ACCM-2) (Fig. 1D), which indicates that *cbu0937* is dispensable for axenic cultivation. Thus, *cbu0937* plays an important role during intracellular replication.

### The gene *cbu0937* has a cell-intrinsic role in supporting intracellular replication of *C. burnetii*

**Fig 2:**
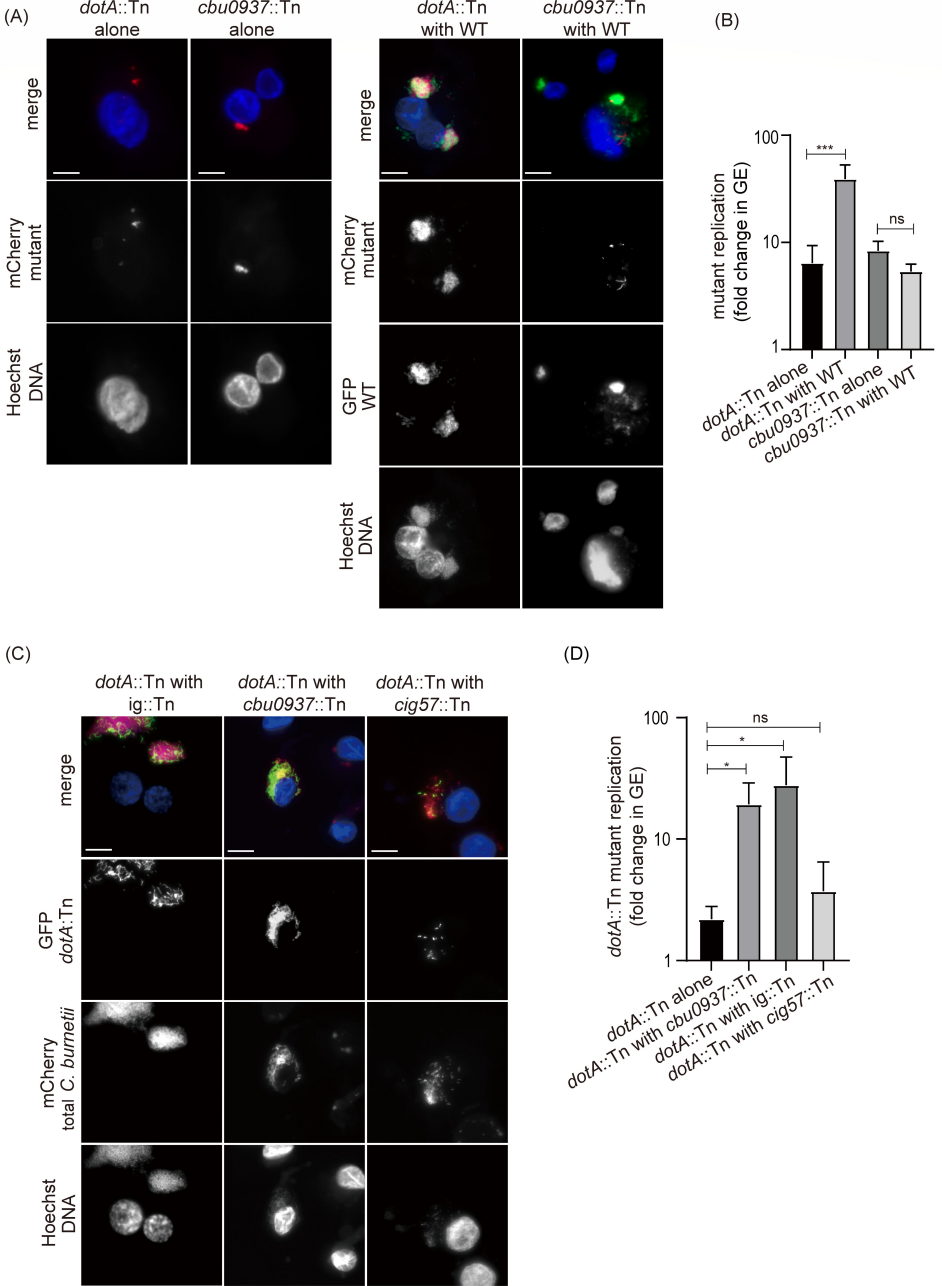
The gene *cbu0937* has a cell-intrinsic role in supporting intracellular replication of *C. burnetii*. (A) Representative immunofluorescence micrographs of THP-1 cells infected with the *dotA*::Tn mutant alone or the *cbu0937*::Tn mutant alone, and cells co-infected with GFP-positive wild-type (WT) *C. burnetii* NMII and the indicated mCherry-expressing transposon mutants. THP-1 cells were infected for 5 days at a multiplicity of infection (MOI) of 2. In the co-infection, cells were infected with a 1:1 mixture of WT and the *dotA*::Tn mutant, or a 1:1 mixture of WT and the *cbu0937*::Tn mutant. Cells were stained with Hoechst 33342 (blue). Scale bars = 10 µm. (B) Quantification of intracellular replication of indicated mutants alone or during a co-infection with WT. THP-1 cells were infected for 5 days at an MOI of 2. GEs were quantified by quantitative PCR (qPCR) assays using a probe specific to *mCherry*. The data shown represent one experiment from three independent experiments, each performed with three biological replicates. For each sample, qPCR was performed in duplicate. Error bars are ± standard deviation. Analysis of variance (ANOVA) was used to compare samples. ***, *P* < 0.001; ns, not significant. (C) Representative immunofluorescence micrographs of THP-1 cells co-infected with a GFP-positive *dotA*::Tn mutant and one of each of the following strains at a 1:1 ratio: ig::Tn, *cbu0937*::Tn, and *cig57*::Tn mutants. Cells were infected for 5 days with mixed cultures at an MOI of 2. Samples were stained with Hoechst 33342 (blue). Scale bars = 10 µm. (D) Quantification of intracellular replication of the GFP-positive *dotA*::Tn mutant alone or during co-infection with either the *cbu0937*::Tn, ig::Tn, or *cig57*::Tn mutant. THP-1 cells were infected for 5 days at an MOI of 2. GEs were quantified by qPCRs using a pair of *GFP*-specific primers. The data shown represent one experiment from three independent experiments, each performed with three biological replicates. For each sample, qPCR was performed in duplicate. Error bars are ± standard deviation. ANOVA was used to compare samples. *, *P* < 0.05; ns, not significant.

Bacterial effectors are defined as proteins that are delivered across the vacuolar membrane to manipulate host processes in the host cell cytosolic environment. As such, effectors function outside the bacterial cell, exhibiting a bacterial cell-extrinsic role. Importantly, it has been shown that strains of *C. burnetii* capable of delivering the full repertoire of effectors can create vacuoles that support intracellular replication of a *C. burnetii* mutant deficient in Dot/Icm function (30), which demonstrates that effectors do not have a bacterial cell-intrinsic role in supporting intracellular replication of *C. burnetii*. Because the Cbu0937 protein was proposed to function as an effector that has a bacterial cell-extrinsic role in mitochondria manipulation, we set out to test this hypothesis by conducting co-infection experiments that examined replication of a *cbu0937*::Tn mutant within vacuoles created by a strain of *C. burnetii* that delivers a full repertoire of effectors, including Cbu0937. To ensure that the different strains could be clearly distinguished using fluorescence microscopy, a wild-type strain producing green fluorescent protein (GFP) and transposon mutants producing mCherry were used in this assay. As expected, a *dotA*::Tn mutant deficient in effector translocation had a severe intracellular replication defect in THP-1 cells (Fig. 2A and B); when the cells were infected with a 1:1 mixture of the *dotA*::Tn mutant and wild-type *C. burnetii*, vacuoles containing the GFP-producing wild-type *C. burnetii* supported replication of the mCherry-producing *dotA*::Tn mutant (Fig. 2A). By contrast, a wild-type co-infection did not rescue the intracellular replication defect of the *cbu0937*::Tn mutant. Very few mCherry-producing *cbu0937*::Tn mutant *C. burnetii* were present in vacuoles created by the replicating GFP-positive wild-type *C. burnetii* (Fig. 2A). These results were independently confirmed by quantifying *C. burnetii* intracellular replication. To differentiate the replication of transposon mutants from wild-type *C. burnetii*, primers for the transposon-encoded *mCherry* gene were used to measure GEs for the mutant bacteria. An increase in GEs of the *dotA*::Tn mutant was observed when the cells were co-infected with the wild-type *C. burnetii* compared to the absence of a co-infection (Fig. 2B). However, GEs of the *cbu0937*::Tn mutant slightly decreased upon co-infection with wild-type *C. burnetii*, implying that the wild-type outcompeted the *cbu0937*::Tn mutant (Fig. 2B). These data indicate that the *cbu0937*::Tn mutant has a bacterial cell-intrinsic defect that prevents efficient replication in the CCV generated by wild-type *C. burnetii*, which suggests that Cbu0937 may not be functioning as a translocated effector.

A corollary to the hypothesis that Cbu0937 has a bacterial cell-intrinsic function required for intracellular replication, independent of Dot/Icm function, is that the cbu0937::Tn mutant should be capable of delivering a full repertoire of effectors and creating CCVs that support replication of a Dot/Icm-deficient mutant. To test this hypothesis, co-infection studies were conducted with a *cbu0937*::Tn mutant and a GFP-producing *dotA*::Tn mutant. Replication of the *dotA*::Tn mutant was quantified by measuring GEs using primers for the *GFP* gene. In these co-infection experiments, robust replication of the *dotA*::Tn mutant was observed in vacuoles generated by the *cbu0937*::Tn mutant (Fig. 2C and D). A mutant with a transposon insertion in *cig57*, which encodes a Dot/Icm effector required for intracellular replication (16), served as a negative control. The *cig57*::Tn mutant was unable to efficiently restore intracellular replication of the *dotA*::Tn mutant (Fig. 2C and D). The vacuoles created by the *cbu0937*::Tn mutant supported similar levels of *dotA*::Tn mutant replication as vacuoles created by the ig::Tn strain (Fig. 2D). Thus, the *cbu0937*::Tn mutant is able to create mature CCVs that support intracellular replication of the *dotA*::Tn mutant, which confirms that the defect of the *cbu0937*::Tn mutant is bacterial cell-intrinsic.

### Structural predictions indicate that *cbu0937* encodes a bacterial porin

**Fig 3:**
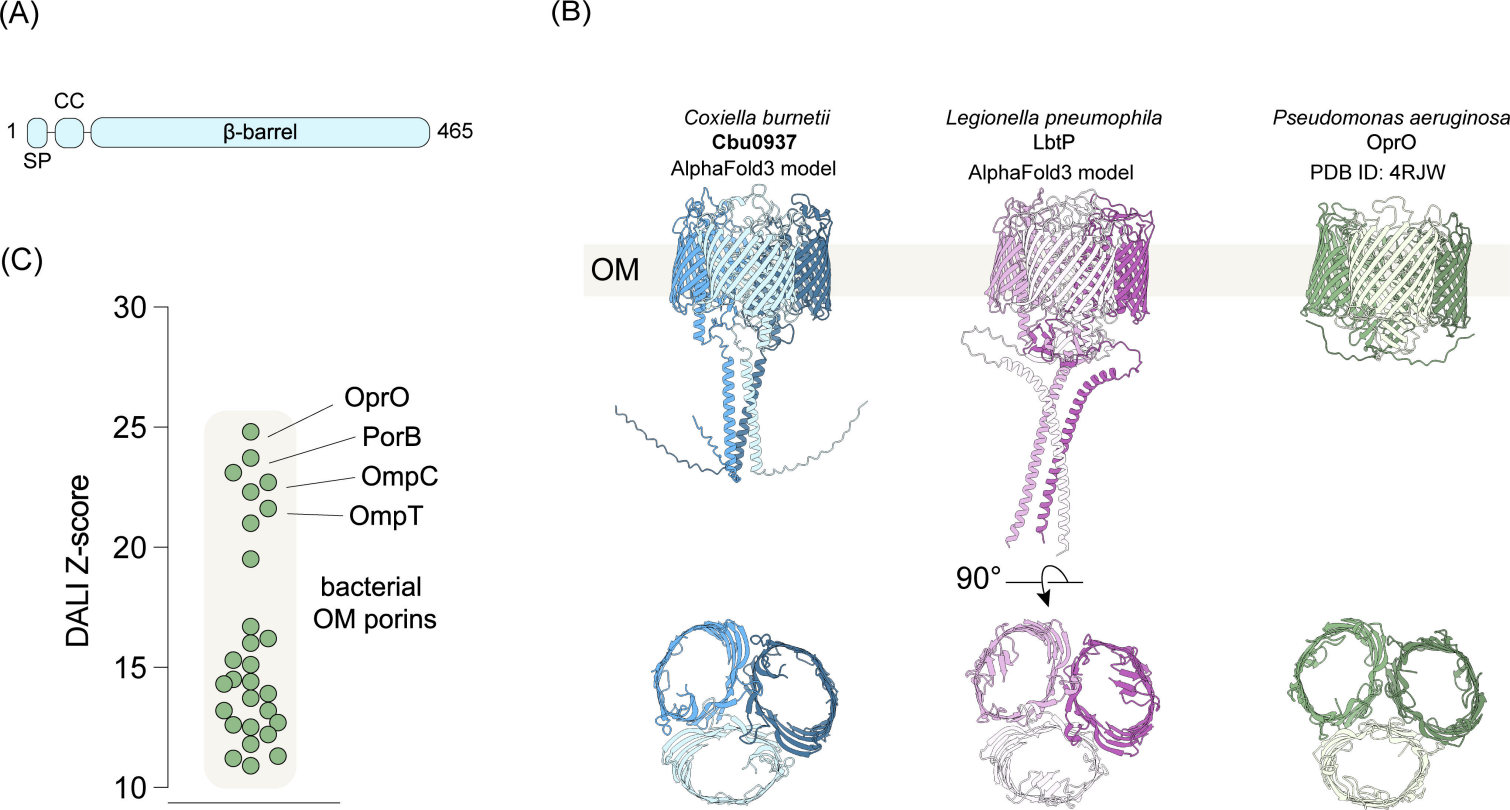
Structural predictions indicate that *cbu0937* encodes a bacterial porin. (A) Schematic depicting Cbu0937 predicted domain architecture. SP, signal peptide; CC, coiled-coil. Length is to scale. (B) AlphaFold3-generated models of Cbu0937 (blue) and *Legionella pneumophila* homolog LbtP (purple) compared to cartoon model of the X-ray crystal structure of the *Pseudomonas aeruginosa* OprO porin (green) demonstrating overall similar structure. Each monomer with the trimers is colored a different shade. Bottom: Cutaway of the β-barrel lumen. (C) Distance matrix alignment (DALI) Z-scores of the top 25 hits from search results of the RCSB PDB25 queried with the Alphafold3 model of Cbu0937. Each point represents a known bacterial outer membrane protein. Representative well-characterized bacterial outer membrane β-barrel proteins are indicated.

To better understand how Cbu0937 supports intracellular replication of *C. burnetii,* the structure of Cbu0937 was modeled with AlphaFold3 (31–33). A high confidence structure consisting of a 16-stranded β-barrel ranging from residue 77 to the C-terminus was predicted (Fig. 3A and B). According to SignalP 6.0 (34), the N-terminal amino acid sequence of Cbu0937 is predicted to harbor a Sec/SPI substrate with the cleavage site between residue 23 and 24 (Fig. 3A). Together, these two analyses suggest that Cbu0937 is a protein that resides in the bacterial outer membrane. A BLAST homology search using the primary amino acid sequence of Cbu0937 identified LbtP of *L. pneumophila* as a potential homolog (Fig. S3). LbtP and Cbu0937 exhibited a similar overall fold, as demonstrated by a low root mean square deviation (RMSD) value of 2.5 Å (<3.0 Å) and a high distance matrix alignment (DALI) Z-score of 51.3 (>20), indicating significant structural similarity between the two proteins (Fig. S4). Comparison of the AlphaFold3 model of Cbu0937 against experimentally determined protein structures from the Protein Data Bank database revealed a structural similarity to bacterial porins residing in the outer membrane (Fig. 3C). Among the top hits, the well-characterized polyphosphate-selective porin OprO of *Pseudomonas aeruginosa*, which shares 10% amino acid identity with Cbu0937, is predicted to be structurally similar to Cbu0937 and LbtP (Fig. 3B). Like OprO, Cbu0937 and LbtP are predicted to form homotrimeric complexes (Fig. 3B; Fig. S5). The predicted Cbu0937 and LbtP structures have extended N-terminal regions with an α-helix that interacts with other monomers (Fig. 3B). Taken together, these *in silico* data indicate that Cbu0937 is a porin that resides in the outer membrane of the bacterial cell.

### The *cbu0937* gene encodes a protein that resides in the outer membrane of *C. burnetii*

**Fig 4:**
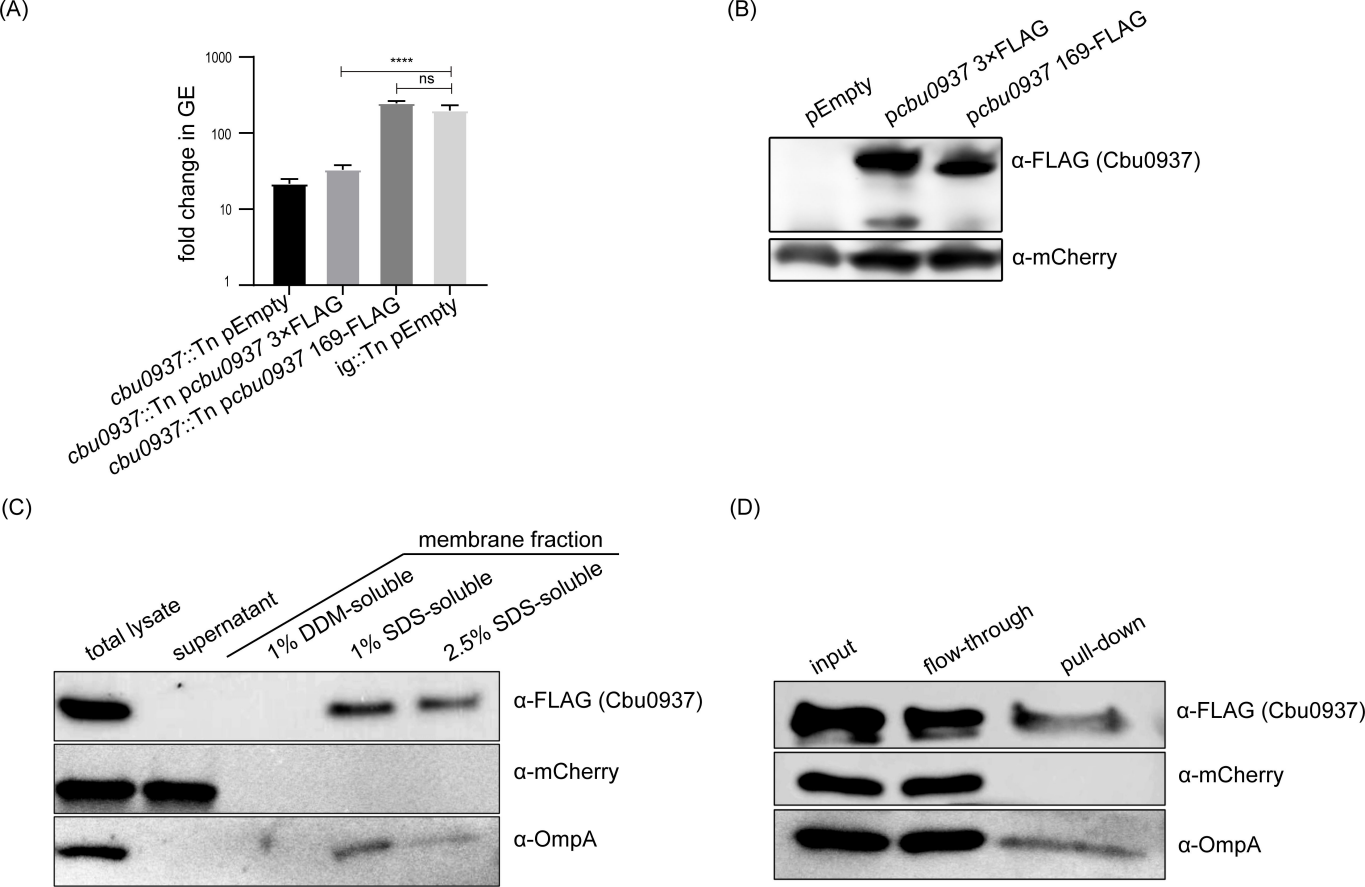
The *cbu0937* gene encodes a protein that resides in the outer membrane of *C. burnetii*. (A) Intracellular replication of the ig::Tn strain harboring an empty vector (pEmpty), the *cbu0937*::Tn mutant harboring an empty vector (pEmpty), the *cbu0937*::Tn mutant harboring a vector encoding Cbu0937 with a C-terminal 3×FLAG tag (p*cbu0937* 3×FLAG), and the *cbu0937*::Tn mutant harboring a vector expressing an internal FLAG-tagged Cbu0937 (Cbu0937 169-FLAG). THP-1 cells were infected for 5 days at a multiplicity of infection of 2. The data shown represent one experiment from three independent experiments, each performed with three biological replicates and two technical replicates. Error bars are ± standard deviation. Analysis of variance was used to compare samples. ****, *P* < 0.0001; ns, not significant. (B) Representative immunoblot analysis of ectopically produced Cbu0937 variants in *cbu0937*::Tn mutant harboring indicated plasmids. Cbu0937 was detected using an anti-FLAG M2 antibody, and an anti-mCherry antibody (ab286186) was used to detect the control mCherry protein. Immunoblot analysis samples were collected from the cultures used in the experiments shown in (**A**), and each sample set was analyzed twice by immunoblot. (C) Representative immunoblot analysis of subcellular fractions of lysed *C. burnetii* producing Cbu0937 169-FLAG. Cbu0937 was detected using an anti-FLAG M2 antibody. Controls for the subcellular fractions were mCherry, detected using an anti-mCherry antibody (ab286186), and the outer membrane protein OmpA, detected using an anti-OmpA antibody (35). The fractionation assay was performed four times independently, and each sample set was analyzed twice by immunoblot. (D) Representative immunoblot analysis of the proteins pulled down from the surface-labeled *C. burnetii*. Surface proteins of *C. burnetii* producing Cbu0937 169-FLAG were labeled with EZ-Link Sulfo-NHS-SS-Biotin. The labeled proteins were pulled down using streptavidin beads. Input refers to the total cell lysate before immunoprecipitation, flow-through refers to the supernatant after immunoprecipitation, and pull-down refers to the bead-bound fraction. Immunoblots were probed as in (**C**). The surface-labeling assay was performed independently three times.

To determine the localization of Cbu0937, we constructed *C. burnetii cbu0937*::Tn mutants producing epitope-tagged Cbu0937 proteins. Placing an epitope tag at the C-terminus of Cbu0937 resulted in the production of a non-functional protein, as determined by an inability to complement the intracellular replication defect of the *cbu0937*::Tn mutant (Fig. 4A). Protein expression was confirmed by immunoblot analysis (Fig. 4B). Structural predictions suggested inserting the FLAG-epitope between amino acids 169 and 170 of Cbu0937 would place the tag in a soluble loop, thus unlikely to disrupt protein function. Indeed, a Cbu0937 variant harboring an internal FLAG tag (Cbu0937 169-FLAG) was stably produced (Fig. 4B) and complemented the intracellular replication defect of the *cbu0937*::Tn mutant (Fig. 4A), indicating that the epitope tag did not disrupt protein function. Fractionation studies were conducted to determine the subcellular localization of Cbu0937. Bacterial lysates were generated from an axenic culture of *C. burnetii* producing Cbu0937 169-FLAG, then proteins were separated into soluble and membrane fractions by ultracentrifugation. The soluble supernatant solution was collected, and the pellet consisting of the bacterial membrane fraction was resuspended in 1% *n*-dodecyl-β-D-maltoside (DDM) to solubilize most inner membrane proteins. The 1% DDM-insoluble pellet was resuspended in 1% SDS to solubilize outer membrane proteins (OMPs), and then, the remaining insoluble proteins were treated with 2.5% SDS buffer to further dissolve β-barrel proteins. The cytosolic mCherry protein was detected only in the supernatant fraction (Fig. 4C), which validates that cytosolic proteins were separated from membrane proteins following ultracentrifugation. The Cbu0937 169-FLAG protein was present only in the membrane fraction (Fig. 4C). Consistent with *cbu0937* encoding a porin, the Cbu0937 169-FLAG protein fractionated similarly to OmpA, a *C. burnetii* outer membrane protein (35). Specifically, Cbu0937 169-FLAG protein was not detected in the 1% DDM-soluble fraction but was present in the 1% SDS-soluble outer membrane fraction and the 2.5% SDS-soluble fraction (Fig. 4C). To independently validate that Cbu0937 and OmpA are surface-exposed proteins, axenically grown *C. burnetii* were biotinylated using the membrane-impermeable EZ-Link Sulfo-NHS-SS-Biotin, and biotin-labeled proteins were isolated in a pull-down after binding to streptavidin beads. The cytosolic mCherry protein was detected only in the flow-through and not in the streptavidin pulled-down fraction (Fig. 4D), which demonstrates that cytosolic proteins were not biotin-labeled. In contrast, FLAG-tagged Cbu0937 and OmpA were pulled down with streptavidin beads (Fig. 4D), indicating that FLAG-tagged Cbu0937 and OmpA were surface-exposed and biotin-labeled. Together, these data confirm the structural predictions indicating that *cbu0937* encodes an outer membrane-localized bacterial porin.

## DISCUSSION

Although many genes have been identified as crucial for CCV biogenesis and intracellular replication (16, 27, 35, 36), the specific roles of most remain unclear. This study confirmed an important role for the gene *cbu0937* in *C. burnetii* pathogenesis by demonstrating that the protein Cbu0937 functions as a porin necessary for efficient intracellular replication of *C. burnetii*.

Many bacterial factors important for intracellular replication have been characterized as Dot/Icm effectors (16, 26, 27, 37, 38). The Dot/Icm type IVB secretion system of *C. burnetii* shares substantial homology with that of the closely related pathogen *L. pneumophila* (39). Notably, multiple *C. burnetii* Dot/Icm genes can complement corresponding mutations in *L. pneumophila* (40, 41), suggesting that *L. pneumophila* could be exploited to dissect the function of the *C. burnetii* secretion system (42). Most *C. burnetii* Dot/Icm effectors were identified through bioinformatic predictions and the use of *L. pneumophila* as a surrogate effector delivery platform (27, 28, 43–45). However, the role of Cbu0937 has been contentious. Although a *L. pneumophila* CyaA translocation assay indicated that Cbu0937 could be translocated into host cells by a Dot/Icm-dependent mechanism (28), a recent study reported that Cbu0937 is not translocated by *C. burnetii* (46). To clarify the role of Cbu0937, we used intravacuolar co-infection assays to determine whether Cbu0937 functions as a Dot/Icm effector. Our findings reveal that the *cbu0937*::Tn mutant has a cell-intrinsic defect. Despite the loss of *cbu0937* function, the *cbu0937*::Tn mutant was still able to form mature vacuoles capable of supporting *dotA*::Tn mutant replication, indicating that Cbu0937 does not promote *C. burnetii* intracellular replication through a bacterial cell-extrinsic effector function.

Bioinformatic analysis was used to further examine Cbu0937 function. The Cbu0937 protein was predicted to consist of a β-barrel with a signal peptide, consistent with previous studies involving *in silico* predictions and mass spectrometry of membrane fractions (47, 48). The β-barrel assembly mechanism is evolutionarily conserved, with mitochondrial β-barrel OMPs capable of integrating into the bacterial outer membrane, and bacterial OMPs expressed in yeast shown to assemble into the mitochondrial outer membrane (49). This evolutionary conservation suggests that ectopic expression of *cbu0937* in host cells may not accurately reflect the biological localization of endogenous *C. burnetii* protein Cbu0937.

To determine the localization of Cbu0937, we constructed epitope-tagged variants of the protein. Initially, a 3×FLAG tag was added to the C-terminus of Cbu0937; however, this modification impaired protein function, as evidenced by the inability of this protein to complement the intracellular replication defect of the *cbu0937*::Tn mutant. This likely reflects the importance of the C-terminal phenylalanine residue, which is essential for proper import and folding of porins in the outer membrane (50). To avoid disrupting function, a FLAG epitope was inserted into what the Cbu0937 structural model predicted would be a flexible loop between two β-sheets within the barrel structure. Fractionation and surface-labeling assays confirmed that the internally FLAG-tagged Cbu0937 protein localized to the bacterial outer membrane. Importantly, although Cbu0937 was reported to be detectable in host cells (29), our data indicated that it does not appear to have an essential function as a translocated effector. Because Cbu0937 was found to be an outer membrane protein, it is reasonable to assume that Cbu0937 would be a constituent of outer membrane vesicles that are shed during *C. burnetii* replication (51), and that proteins in outer membrane vesicles would be present in host cell fractions after bacterial cells are pelleted after infection. This could account for the detection of Cbu0937 in host cells and possible co-fractionation with host cell mitochondria from infected cells. In summary, our study demonstrates that Cbu0937 is a bacterial outer membrane protein essential for intracellular replication of *C. burnetii*. Given this functional role, we propose renaming *cbu0937* as *ompI* (outer membrane protein for intracellular replication).

The outer membrane localization and the porin-like structure of Cbu0937 suggest that this porin is important for acquisition of a nutrient present in the lumen of the CCV. One possibility is that Cbu0937 functions in iron uptake. This hypothesis is based on the observation that the *L. pneumophila* outer membrane protein LbtP, which is involved in iron uptake, is predicted to be a homolog of Cbu0937. Loss of LbtP impairs iron acquisition and restricts *L. pneumophila* replication in macrophages (52). *C. burnetii* requires iron to support optimal replication and viability (53), yet the mechanisms of iron acquisition by *C. burnetii* are poorly understood. Genomic analyses suggest that iron uptake is mediated by the FeoAB transporter in the inner membrane (53, 54), but the corresponding outer membrane transporter remains unidentified. Whether Cbu0937 serves as an outer membrane iron importer for *C. burnetii* requires additional studies that would provide crucial insights into intracellular nutrient acquisition strategies used by this obligate intracellular pathogen.

## MATERIALS AND METHODS

### *C. burnetii* strains and mammalian cell lines

Bacterial strains used in this study are listed in Table S1. *C. burnetii* strains were grown in ACCM-2 at 37°C, in 5% CO_2_ and 2.5% O_2_ as described (55, 56). Media were supplemented with 3 µg/mL chloramphenicol (transposon mutants) and/or 375 µg/mL kanamycin (plasmid maintenance) where necessary. THP-1 cells and HeLa cells (HeLa, ATCC CCL-2) were bought from ATCC. THP-1 cells were cultured in Roswell Park Memorial Institute (RPMI) 1640 Medium (ATCC modification, catalog no. A10491-01) supplemented with 10% heat-inactivated fetal bovine serum (FBS) at 37°C in 5% CO_2_. Prior to infections, THP-1 cells were differentiated with 125 ng/mL phorbol 12-myristate 13-acetate for 2 days. HeLa cells were grown in Dulbecco’s Modified Eagle’s Medium supplemented with 10% FBS at 37°C in 5% CO_2_. For assays involving HeLa cells, cells were seeded the day before infection.

### Plasmids and *C. burnetii* transformations

Plasmids used in this study are listed in Table S1. Plasmids were constructed with Gibson assembly (NEB Gibson Assembly Master Mix) and restriction enzyme cloning (restriction enzyme: SalI, PstI-HF, EcoRI). Plasmids were introduced into *C. burnetii* via electroporation with an Electro Cell Manipulator ECM630 (BTX Harvard Apparatus) (settings: 1.8 kV, 500 Ω, 25 µF) as previously described (16, 36). Following electroporation, *C. burnetii* were recovered in 3 mL of ACCM-2 for 24 h before the appropriate antibiotic was added. Following a further 4 days, *C. burnetii* were plated on ACCM-2 agarose plates containing selective antibiotics. After 10 days of incubation, single colonies were isolated and expanded in liquid ACCM-2. The presence of plasmids and the expression of desired proteins were confirmed by PCR and immunoblot analysis, respectively.

### *C. burnetii* infections

*C. burnetii* cultures were centrifuged at 3,000 *g* for 15 min, then cells were resuspended in RPMI containing 10% FBS and sonicated for 10 min in an ultrasonic water bath. *C. burnetii* GEs were measured by quantitative PCR (qPCR) using *dotA* primers as described previously (16). Differentiated THP-1 cells were infected with *C. burnetii* at a multiplicity of infection (MOI) of 2. HeLa cells were infected at an MOI of 50. For co-infections, corresponding strains were mixed at a 1:1 ratio. Approximately 2 h after infection, cells were washed three times with Dulbecco's Phosphate Buffered Saline (DPBS) and incubated with fresh medium. Lysates of infected cells were collected at defined time points post-infection.

### GE quantifications in infected cells

Infected cells were lysed with sterile, nuclease-free deionized water. Lysates were centrifuged at 21,000 *g* for 5 min, then resuspended in 150 µL of nuclease-free H_2_O and 100 µL of glass beads as described (57). Bacteria were lysed with a Next Advance BBX24 bullet blender (settings: speed 8, 5 min). The samples were briefly centrifuged and then boiled for 10 min. After boiling, the beads and cell debris were pelleted by centrifugation. Approximately 80 µL of supernatant was transferred to a clean tube. GEs were quantified by qPCRs using probes specific to *dotA*. In co-infection assays, GEs of strains expressing mCherry or GFP were quantified using probes specific to *mCherry* or *GFP*.

### Immunofluorescence microscopy

At 5 days post-infection, cells were fixed with 4% paraformaldehyde in DPBS for 15 min at room temperature and washed three times with DPBS. The samples were permeabilized and blocked for 1 h with 5% goat serum plus 0.1% saponin at room temperature. Antibodies were diluted in DPBS containing 0.1% saponin and 1% bovine serum albumin (BSA). The cells were stained with primary antibodies for 1 h at room temperature. Rabbit anti-*C*. *burnetii* (1:20,000) (58) and mouse anti-LAMP-1 (1:700) (H4A3-c, Developmental Studies Hybridoma Bank, University of Iowa) antibodies were used as primary antibodies. The samples were rinsed with DPBS three times, then incubated with diluted secondary antibodies for 1 h. Invitrogen secondary antibodies, Alexa Fluor goat anti-mouse 488 (1:500) and Alexa Fluor goat anti-rabbit 568 (1:500), were used. 4′,6-diamidino-2-phenylindole (DAPI, 0.2 µg/mL, Sigma) or Hoechst 33342 (1 μg/mL, Thermo Fisher Scientific) was added during DPBS washes after secondary antibodies incubation to stain bacterial and host DNA. Coverslips were mounted on slides using ProLong Gold Antifade Mountant (Invitrogen). Images were acquired using a Nikon Eclipse TE2000-S inverted fluorescence microscope equipped with a Nikon 100×/1.4 numerical aperture objective lens. Images were analyzed in SlideBook software (Intelligent Imaging Innovations) and Fiji (ImageJ).

### Structural modeling and analysis of Cbu0937

The amino acid sequence of Cbu0937 from the *C. burnetii* RSA439 genome (Genbank accession number CP020616.1) was used as the query in BLASTp search and SignalP 6.0 prediction (34). The predicted structure of Cbu0937 and LbtP was generated using AlphaFold3 (31). The DALI server was used to compare the structure of Cbu0937 against a representative subset of the Protein Data Bank (59). Structure analysis was conducted in PyMOL (Schrödinger, LLC) and Chimera.

### Bacterial fractionation and immunoblotting

The *C. burnetii* fractionation procedure was modified from a fractionation protocol for *L. pneumophila* (60). *C. burnetii* strains were grown in ACCM-2 for 6 days. *C. burnetii* cultures were diluted 1:100 into 80 mL ACCM-2 and incubated for 4 additional days. The following steps were performed at 4℃. Cells were pelleted, and the pellet was resuspended in 2 mL of 250 mM sucrose and 50 mM Tris-HCl, pH 8.0. Then, 10 µL of 0.5 M EDTA (pH 8.0) and 10 µL of 10 mg/mL hen egg white lysozyme were added and incubated on ice for 30 min before the addition of 40 µL of 1 M MgSO_4_. The sample was centrifuged for 5 min at 5,000 *g*, then the pellet was resuspended in 1 mL of 50 mM Tris-HCl, pH 8.0, with protease inhibitor (cOmplete, Mini, EDTA-free Protease Inhibitor Cocktail) and glass beads. A Next Advance BBX24 bullet blender was used at speed 8 for 10 min to lyse the bacteria. Glass beads and unlysed cells were removed by centrifugation at 5,000 *g* for 10 min. The supernatant was the total cell lysate containing soluble cytosolic proteins and membrane-associated proteins. Total membranes were isolated from these lysates by centrifugation at 55,000 *g* for 1 h. The membrane pellet was resuspended in 900 µL of 50 mM Tris-HCl (pH 8.0), and 100 µL of 10% DDM was slowly added into the suspension to dissolve the inner membrane. The suspension was centrifuged again for 1 h at 55,000 *g*, and the pellet containing outer membrane proteins was resuspended in 250 µL of 50 mM Tris-HCl (pH 8.0) containing 1% SDS. After a final centrifugation at 55,000 *g* for 1 h, the 1% SDS-insoluble pellet was resuspended in 100 µL of 50 mM Tris-HCl (pH 8.0) with SDS-PAGE sample buffer containing 2.5% SDS and 8M urea. The loading amount was adjusted using sample buffer. All samples were boiled in SDS-PAGE sample buffer with urea for 10 min and loaded onto a 15% SDS-PAGE gel. Immunoblotting was performed as previously described (61). Proteins were detected using mouse anti-FLAG (1:1,000), rabbit anti-mCherry (1:500), and rabbit anti-OmpA (1:150) primary antibodies and horseradish peroxidase (HRP)-conjugated goat secondary antibodies (Invitrogen).

### Bacterial surface labeling and pull-down

The surface-labeling protocol was adapted from a published method for biotinylation and purification of surface-exposed *Helicobacter pylori* proteins (62). *C. burnetii* producing Cbu0937-169-FLAG were grown in ACCM-2 for 5 days. Cultures were diluted 1:100 into 100 mL of fresh ACCM-2 and incubated for an additional 4 days. Bacteria were pelleted by centrifugation and resuspended in biotinylation buffer (1× phosphate-buffered saline (PBS), 1 mM CaCl_2_, 0.5 mM MgCl_2_, 1.6 mM D-biotin, pH 7.4), followed by a wash with the same buffer. EZ-Link Sulfo-NHS-LC-Biotin (Thermo Fisher Scientific) was added to a final concentration of 200 µM. The reaction was incubated for 30 min on ice and subsequently terminated by adding a quenching buffer (50 mM Tris pH 7.4, 100 mM NaCl, 27 mM KCl, 1 mM CaCl_2_, 0.5 mM MgCl_2_). Bacteria were then washed four times with the quenching buffer and resuspended in 1 mL of lysis buffer (50 mM Tris, pH 7.4, 2 mM EDTA, 1% SDS, 1 mg/mL lysozyme, DNase I, and Complete Mini protease inhibitor) with glass beads. *C. burnetii* were lysed using a Next Advance BBX24 bullet blender at speed 8 for 10 min. Unlysed cells and glass beads were removed by centrifugation. Dynabeads M-280 Streptavidin beads were washed three times and blocked with BSA before use. Bacterial lysates were incubated with streptavidin beads for 3 h at 4℃. After five washes, bound proteins were eluted by boiling in SDS sample buffer and analyzed by immunoblotting.

### Statistical analysis

One‐way analysis of variance or unpaired Student’s *t*-test were conducted using Prism9 (GraphPad Software, Inc.) and Minitab21 (Minitab, LLC).

## SUPPLEMENTAL MATERIAL

Supplemental material iai.00448-24-s0001.pdf Table S1; Fig. S1 to S5.

ASM does not own the copyrights to Supplemental Material that may be linked to, or accessed through, an article. The authors have granted ASM a non-exclusive, world-wide license to publish the Supplemental Material files. Please contact the corresponding author directly for reuse.
