# Supplementary material for: Identification of a Coxiella burnetii outer membrane porin required for intracellular replication
Source: Infect Immun. 2025 Mar 12;93(4):e00448-24. doi: 10.1128/iai.00448-24 (PMC11977307; doi:10.1128/iai.00448-24)
Supplement: Supplemental material — Table S1; Fig. S1 to S5. [file iai.00448-24-s0001.pdf]

Table S1 Strains and plasmids used in this study

***Coxiella burnetii* strains**			
Name	Relevant genotype	Description/comments	Source/ reference
RSA439	*Coxiella burnetii* wild-type (WT)	Plaque-purified Nine Mile phase II (NMII) clone 4	(1)
intergenic::Tn (ig::Tn)	intergenic::TnA7	Transposon insertion mutant between *cbu2077* and *cbu2078*, CmR, mCherry; shows intracellular replication comparable to wild-type *C. burnetii* NMII	(2)
cbu0937::Tn	*cbu0937*::TnA7	Transposon insertion mutant in *cbu0937*, CmR, mCherry	(2)
cig57::Tn	*cbu1751*::TnA7	Transposon insertion mutant in *cbu1751*, CmR, mCherry	(2)
dotA::Tn	*dotA*::TnA7	Transposon insertion mutant in *dotA* (*cbu1648*), CmR, mCherry	(2)
RSA439 GFP	attTn7::miniTn7T-CAT-L3S2P21::P*311*-GFP	WT NMII RSA439 with single copy genomic insertion at attTn7 site expressing GFP under the control of the *cbu0311* promoter, CmR (SS400)	Constructed and gifted by Samuel Steiner, Roy Laboratory, Yale University

dotA::Tn pGFP	*dotA*::TnA7 pJB-Kan-P*311*-GFP	Transposon insertion mutant in *dotA* (*cbu1648*), CmR, mCherry; harboring RSF1010 *C. burnetii* expression vector expressing GFP under the control of the *cbu0311* promoter, KmR (pSAST57)	(3)
***Escherichia coli* strain**			
Name	Relevant genotype	Description/comments	Source/reference
Mach1	str. W Δ*recA1398 endA1 fhuA* Φ80Δ(*lac*)M15 Δ(lac)*X74* hsdR($r_K^- m_{Ks}^+$)	Used for plasmid constructions and maintenance	Invitrogen
**Plasmids**			
Name	Relevant genotype	Description/comments	Source/reference
pJB-Kan	KmR AmpR	RSF1010 *C. burnetii* expression vector (*cbu1169* promoter)	(4)
pJB-Kan-3×F	KmR AmpR	RSF1010 *C. burnetii* expression vector for 3×FLAG fusions (*cbu1169* promoter)	(4)
pJB-Kan-*cbu0937*	KmR AmpR, *cbu0937*	*cbu0937* cloned into pJB-Kan	This study
pJB-Kan-	KmR AmpR, *cbu0937* with	*cbu0937*-3×FLAG; *cbu0937* cloned	This study

cbu0937-3×F	a C-terminal 3×FLAG tag	into pJB-Kan-3×FLAG	
pJB-Kan-*cbu0937* 169-FLAG	KmR AmpR, *cbu0937* with an internal FLAG tag	*cbu0937* with a FLAG tag inserted between 169N and 170P, cloned into pJB-Kan	This study

References

1. **Williams JC, Peacock MG, McCaul TF.** 1981. Immunological and biological characterization of Coxiella burnetii, phases I and II, separated from host components. Infect Immun **32:**840-851.
2. **Newton HJ, Kohler LJ, McDonough JA, Temoche-Diaz M, Crabill E, Hartland EL, Roy CR.** 2014. A screen of Coxiella burnetii mutants reveals important roles for Dot/Icm effectors and host autophagy in vacuole biogenesis. PLoS Pathog **10:**e1004286.
3. **Steiner S, Meir A, Roy CR.** 2021. Coxiella burnetii encodes an LvgA-related protein important for intracellular replication. Cell Microbiol **23:**e13331.
4. **Beare PA.** 2012. Genetic manipulation of Coxiella burnetii. Adv Exp Med Biol **984:**249-271.

FIG S1

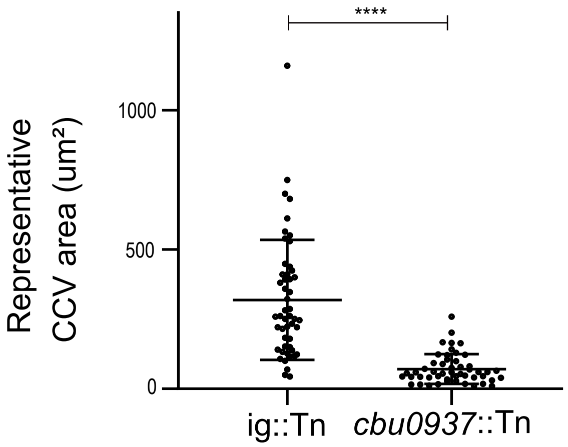

Figure S1 Quantification of CCV size during the infection shown in Fig. 1B
Quantification of the area of CCVs formed by the *cbu0937*::Tn mutant or the ig::Tn strain during the infection demonstrated in Fig. 1B. Error bars are ± standard deviation. 50 CCVs per strain were measured from one representative experiment. Statistical significance was determined using a two-sample t-test. ****, $p < .0001$.

FIG S2

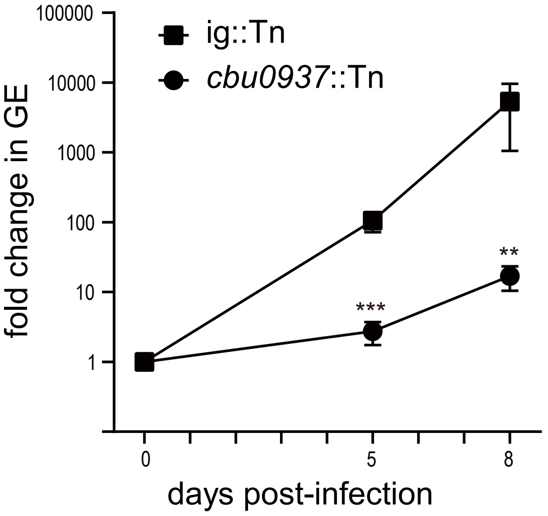

Figure S2 *cbu0937* is necessary for intracellular replication in HeLa cells
Intracellular replication over 8 days in HeLa cells of the *cbu0937*::Tn mutant and the ig::Tn strain quantified by GE. Error bars are ± standard deviation. Statistical significance was determined using Student's t-test. Represented p values are relative to the strain ig::Tn. **, $p < .01$; ***, $p < .001$.

FIG S3

LbtU family siderophore porin [Legionella pneumophila]
Sequence ID: **HAT7923477.1** Length: 530 Number of Matches: 1
Range 1: 1 to 530

Score	Expect	Method	Identities	Positives	Gaps	Frame
272 bits(696)	2e-81()	Compositional matrix adjust.	176/536(33%)	264/536(49%)	77/536(14%)	

```
Query  1     MTSKLVISALGLCVSGALSTTLASTPATTNQQITKRIDYLQAQINELR--------TQQK  52
             M  K +I AL  C+S  L         +  Q++  + + LQAQ+N L+        +QQK
Sbjct  1     MKFKKIILALA-CLSSPLYADQDQQLKSEIQRLQHQAEDLQAQLNRLQKQLANHKSSQQK  59

Query  53    KERQKKKAPYRKCSKKSCK--------YHSSRLSI-------------------------  79
              E+Q    P    SK + K        YHSS++ +
Sbjct  60    HEQQAATKPAEPQSKPTVKSGAAIEEKYHSSKVEVHAPDAHPESISFYPTALIADNRVVT  119

Query  80    ---------GPYLHKTPAFDGSDLIINVPTVREDARLLLLQHQLEEECRALGVPLPEMPR  130
                       P+L   PAFDGSD I+N+ ++  D RLL  + +L    + +G P+P MP
Sbjct  120   YIAGTPVVSSPFLGDRPAFDGSDYIVNISSINRDVRLLQQRRRLYRAYQKIGYPIPNMPI  179

Query  131   VVFSGKLEGQTSYGSTYAGSR-NANINFSGAEFDTYVQANPWVSGYMALDYDPDELADGS  189
             +  SGK E   ++ + +  +  + +I    +E D     N  V  Y+A+ YD    A G
Sbjct  180   ISLSGKTEPAATFNNPFRSTNTDGDITLGSSELDVAAALNENVEAYIAIAYDESPPAIGP  239

Query  190   RVF-----MNRAFIMIGNLSRFPFYTSIGQVYVPFGRYSSMMITTPVTQALGRTRARAIT  244
             RV      +N  F+ IGNL + P Y + GQVYVPFGRYSS M+++PVT  L RT+ R +
Sbjct  240   RVNNSAFNLNMGFVNIGNLDKSPLYFTAGQVYVPFGRYSSAMVSSPVTMNLARTKTRPVI  299

Query  245   LGYQQTGNNALHAELYGYQGLTNNFSRSNHNDQWGTDVGYEFSNGDRVSGEIGASFISNL  304
              GY+  G+      +YGY+  T    RS      G ++GY F   + ++GEIG  FIS++
Sbjct  300   FGYKSQGDTGPFGAVYGYRSDTT-LGRSGVG---GVNLGYIFGFDNDINGEIGGGFISSV  355

Query  305   ADSQGMQATA-------------FLDNETLRHRVSALGVYGSLAIKPVVFIAEYISALKS  351
             AD+ GMQ+T                 NE +R +  A  V+G +         E++ A++S
Sbjct  356   ADAGGMQSTGSNVGTTFGGFGSITNGNENVR-KTKAADVHGHVGYDRYNLTLEWVGAVQS  414

Query  352   FDINDVNFANRGARPTAFHTEANYTFKTGSKPSSIGIGYGHTSQALGVGLPQDRYSVFYN  411
             F   D++F  +GARP A   E   TF   ++P+SIG+GY  T +AL + LP+ RY   +N
Sbjct  415   FRPQDLSFNGQGARPQAAQAELGMTFMAFNRPASIGVGYQWTKEALALNLPKRRYVGVFN  474

Query  412   VNIWKDTNFALEYRHDVNYTRNAISTGTNPTPAKVVADL--GKSDNVVTAQFDLYF  465
             ++IWKDT  ++EYRHD++Y     + G  P     +  L  GKS + V+AQ  +YF
Sbjct  475   ISIWKDTVESIEYRHDIDYGLTQFANGAAPQGFVNLPTLGTGKSADTVSAQIGVYF  530
```

Figure S3 BLASTp alignment of Cbu0937 and LbtP
Sequence alignment between Cbu0937 and LbtP (LbtU family siderophore porin). The alignment highlights regions of sequence similarity, with identical residues marked by letter and positive matches indicated by plus signs (+). The alignment score and E-value are provided to demonstrate the statistical significance of the match. Gaps introduced to optimize the alignment are represented by dashes (-).

FIG S4

Summary

No: Chain Z rmsd lali nres %id Description

1: t001-A 51.3 2.5 382 530 35 MOLECULE: UPF0422 PROTEIN LPG2959

Pairwise Structural Alignments

```
DSSP  --------------------------lllllllllllllllllllllllllhhhhhhh
Query --------------------------mtsklvisalglcvsgalsttlastpattnqqit   34
ident 
Sbjct mkfkkiilalaclssplyadqdqqlkseiqrlqhqaedlqaqlnrlqkqlanhkssqqkh   60
DSSP  llhhhhhhhhhlllllllllllhhhhhhhhhhhhhhhhhhhhhhhhhhhhhhhhhhhlll


DSSP  hhhhhhhhhhhhhhhhhhhhhhhhllllllllLLLL-----------------------lL
Query kridylqaqinelrtqqkkerqkkkapyrkcSKKS-----------------------cK   71
ident 
Sbjct eqqaaakpaepqskptvksgaaieekyhsskVEVHapdahpesisfyptaliadnrvvtY  120
DSSP  lllllllllllllllllllllllllllllllLEELlllllhhhllllllleeeelleeeeE


DSSP  LLLLLEEELLLLLLLLlLLHH-HLLLLLLLLLHHHHHHHHHHHHHHHHHHHLLLLLLLLE
Query YHSSRLSIGPYLHKTPaFDGS-DLIINVPTVREDARLLLLQHQLEEECRALGVPLPEMPR  130
ident          | |   |      | | |      | ||      |       | | | ||
Sbjct IAGTPVVSSPFLGDRP-AFDGsDYIVNISSINRDVRLMQQRRRLYRAYQKIGYPIPNMPI  179
DSSP  LLLLLEEELLLLLLLL-LLLLlLLHHHLLLLLHHHHHHHHHHHHHHHHHHHLLLLLLLLE


DSSP  EEEEEEEEEEEEEEELLLL-LEEEEEEEEEEEEEEEEEELLLLEEEEEEEEEELLL-----l
Query VVFSGKLEGQTSYGSTYAG-SRNANINFSGAEFDTYVQANPWVSGYMALDYDPD-----e  184
ident    ||| |                 |     | |     |  |  | |  ||
Sbjct ISLSGKAEPAATFNNPFRStNTDGDITLGSSELDVAAALNENVEAYIAIAYDESppaigp  239
DSSP  EEEEEEEEEEEEEEELLLLlLLEEEEEEEEEEEEEEEEEELLLLEEEEEEEEEELLLllllll


DSSP  lllLLLEEEEEEEEEEEELLLEEEEEEEEEEELLLLLLLLLLLLHHHHHHLLEEEEEE
Query ladGSRVFMNRAFIMIGNLSRFPFYTSIGQVYVPFGRYSSMMITTPVTQALGRTRARAIT  244
ident     |    |  |  ||||   | |   |||||||||||| |   |||  | ||  |
Sbjct rvnNSAFNLNMGFVNIGNLDKSPLYFTAGQVYVPFGRYSSAMVSSPVTMNLARTKTRPVI  299
DSSP  hhhLLLEEEEEEEEEEEELLLLLLEEEEEEEEEELLLLLLLLLLLLHHHHHHLLEELLEE


DSSP  EEEELLLLEEEEEEEEEEEELLLEELllllLLEEEEEEEEEEEEELLLEEEEEEEEEELLH
Query LGYQQTGNNALHAELYGYQGLTNNFsrsnHNDQWGTDVGYEFSNGDRVSGEIGASFISNL  304
ident  ||            |||   |            |   || |       || |  |||
Sbjct FGYKSQADTGPFGAVYGYRSDTTLG----RSGVGGVNLGYIFGFDNDINGEVGGGFISSI  355
DSSP  EEEELLLLLEEEEEEEEEEELLLLLL----LLEEEEEEEEEEEEELLLEEEEEEEEEELLH


DSSP  HHLHHHHLL---------LLLL----LLEElLLLLEEEEEEEEEELLEEEEEEEEEELLL
Query ADSQGMQAT---------AFLD----NETLrHRVSALGVYGSLAIKPVVFIAEYISALKS  351
ident ||  ||| |          |      ||       |  | |           |   |  |
Sbjct ADAGGMQSTganvgttfgGFGSitngNENV-RKTKAADVHGHVGYDRYNLTLEWVGAIQS  414
DSSP  HHLHHHHLLlllllllllLLLLllllLLLL-LLLLEEEEEEEEEELLEEEEEEEEEELLL


DSSP  LLLLLLEELLEELLLEEEEEEEEEEEEEELLEEEEEEEEEEEEEELLHHHLLLLEEEEEEEE
Query FDINDVNFANRGARPTAFHTEANYTFKTGSKPSSIGIGYGHTSQALGVGLPQDRYSVFYN  411
ident |   |  |   |||| |   |   ||     | ||| ||  |  ||   ||  ||    |
Sbjct FRPQDLSFNGQGARPQAAQAELGMTFMAFNRPASIGVGYQWTKEALALNLPKRRYVGVFN  474
DSSP  LLLLLEELLEELLLEEEEEEEEEEEEEELLEEEEEEEEEEEEEELLHHHLLLLEEEEEEEE


DSSP  EEEELLEEEEEEEEEEEELLLHHHL------lllLLLLlhhHHHHlLLEEEEEEEEEEEEL
Query VNIWKDTNFALEYRHDVNYTRNAI------stgTNPTpakVVADlGKSDNVVTAQFDLYF  465
ident   |||||    |||||  |               |          |||   | ||   ||
Sbjct ISIWKDTVESIEYRHDIDYGLTQFangaapqgfVNLP---TLGT-GKSADTVSAQIGVYF  530
DSSP  ELLLLLEEEEEEEEEEEEELLLLLLEeeellllllLLLL---EELL-LLEEEEEEEEEEEEL
```

Figure S4 Pairwise structural alignment between Cbu0937 and LbtP
Structural alignment between Cbu0937 and LbtP generated using DALI server. The three-state secondary structure defined by DSSP and simplified to H (helix), E (sheet), and L (coil), are displayed above the amino acid sequence. Structurally equivalent residues are presented in uppercase, while structurally non-equivalent residues are shown in lowercase. Vertical bars indicate identical amino acids.

FIG S5

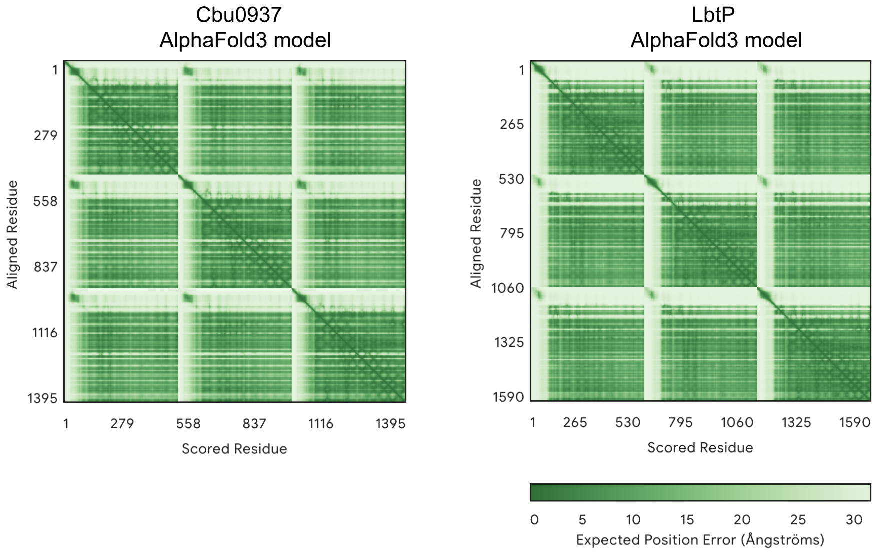

Figure S5 Predicted aligned error plot of AlphaFold3 models
Predicted aligned error plot of AlphaFold3-generated Cbu0937 and LbtP trimers demonstrating high confidence in residue placement within the β-barrel and trimeric structure. Plot indicates per-residue relative position with expected positional error for each residue plotted on the x axis if the structure was aligned on the indicated residue on the y axis. Dark green indicates 0 Å of error and white indicates >30 Å of error.
